# Impact of Identification and Treatment of Depression in Heart Transplant Patients

**DOI:** 10.1155/2014/747293

**Published:** 2014-09-14

**Authors:** Ike Okwuosa, Dara Pumphrey, Jyothy Puthumana, Rachel-Maria Brown, William Cotts

**Affiliations:** ^1^Northwestern Memorial Hospital, Feinberg School of Medicine Department of Medicine, 251 E Huron, Northwestern University, Chicago, IL 60616, USA; ^2^Feinberg School of Medicine Department of Psychiatry, Northwestern University, Chicago, IL 60616, USA; ^3^Feinberg School of Medicine Department of Medicine, Division of Cardiology, Northwestern University, Chicago, IL 60616, USA

## Abstract

*Background*. The effects of clinical depression after orthotopic heart transplantation (OHT) are relatively unknown. The purpose of this study was to evaluate the impact of depression on outcomes after OHT. *Methods*. We performed a single center retrospective review of 102 consecutive patients who underwent OHT at Northwestern Memorial Hospital from June 2005 to October 2009. The diagnosis of depression was obtained from attending physician documentation. The primary endpoints were all-cause mortality (ACM), hospitalizations, and rejection. *Results*. Of 102 OHT patients, 26 (26%) had depression. Depressed patients were similar in age to nondepressed patients (57.6 years versus 56.9, *P* = 0.79). There was no statistical difference in survival between groups at 5 years after OHT (*P* = 0.94). All-cause hospitalizations were higher in depressed versus nondepressed patients (4.3 versus 2.6 hospitalizations *P* = 0.05). There were no significant differences in hospitalizations between the two groups for the following complications: cardiac (heart failure, edema, arrhythmias, and acute rejection) and infections. There was no significant difference in episodes of 2R and 3R rejection. *Conclusion*. Early identification and treatment of depression in OHT patients result in outcomes similar to nondepressed patients.

## 1. Introduction

Heart failure is associated with significant resource utilization, accounting for nearly 1 million hospital admissions annually, placing a significant financial burden on the healthcare system [1]. In 2010 $24 billion were dedicated to care for heart failure patient; by 2030 that cost is projected to total $70 billion [2]. Similar to other chronic diseases such as diabetes mellitus and chronic obstructive pulmonary disease, depression affects an estimated 20% of heart failure patients [3–5], compared to the estimated 6.6% amongst the general population [6].

OHT is definitive and the best treatment option available for patients with end stage heart failure. Following heart transplantation patients report improved functional capacity and overall quality of life; however this varies based on severity of illness before transplant [7, 8]. A significant portion of patients after OHT experience depression (estimated prevalence of 20–30%) [9, 10].

Independently, depression has been associated with increased resource utilization. It is estimated that depression is responsible for $34 billion in direct and indirect costs annually [11]. In patients with chronic diseases, depression has been associated with increased morbidity and mortality, when compared to nondepressed patients [12, 13]. Depression has also been associated with medication nonadherence and increased hospitalizations. In a patient population with OHT, this could have serious adverse outcomes and represents a serious challenge in posttransplant management.

Studies have shown that early identification and initiation of treatment for depression are associated with improved outcomes [14]. Antidepressant medication represents the best established treatment for patients with a diagnosis of depression [15]. So it can be implied that early identification and treatment of depression in OHT patients should lead to favorable outcomes.

Current studies evaluating depression and its effects in the OHT patient population have focused primarily on quality of life [16–20]. To our knowledge, no study to date has evaluated the impact of early identification and treatment of depression on survival, rehospitalizations, and organ rejection.

## 2. Methods

### 2.1. Study Design

The study was reviewed and approved by the internal review board. This study was performed as a single center retrospective electronic medical record (EMR) review of consecutive patients who underwent OHT at Northwestern Memorial Hospital from June 2005 through October 2009. The* Diagnostics and Statistical Manual Fourth Edition-Text Revision* (DSM-IV-TR) defines major depressive disorder as “a mood disorder having a clinical course involving one or more episodes of serious psychological depression that last two or more weeks each, do not have intervening episodes of mania or hypomania, and are characterized by a loss of interest or pleasure in almost all activities and by some or all of disturbances of appetite, sleep, or psychomotor functioning, a decrease in energy, difficulties in thinking or making decisions, loss of self-esteem or feelings of guilt, and suicidal thoughts or attempts.” For the purpose of our study we selected patients who were diagnosed as having depression as verified by attending physician documentation in the medical record. We did not utilize the DSM-IV-TR criteria for major depressive disorder. Using stringent criteria as this would limit our study as this information was not readily available in our medical records. This would have also resulted in a less inclusive study that would ultimately be less generalizable, as many individuals with medical illness suffer from some depressive symptoms and do not meet criteria for major depressive disorder. Making the diagnosis of major depressive disorder can also be challenging in individuals with chronic illnesses as some of the symptoms of major depressive disorder (e.g., decreased appetite and decreased energy) overlap with symptoms of chronic illnesses themselves. All patients underwent pretransplantation evaluation by the Cardiovascular Behavioral Medicine team, and depressed patients were further evaluated by the inpatient Psychiatric Liaison service. The retrospective nature of this study limited the availability of severity of depression. Inpatient and outpatient medical records were reviewed for antidepressant medications. Baseline characteristics including the prior use of mechanical circulatory assist devices were obtained. Complete follow-up information including total hospitalizations subcategorized into cardiac and infectious hospitalizations was obtained. Cardiac hospitalizations included hospitalization for arrhythmias, acute coronary syndrome, chest pain, shortness of breath, cardiac rejection, and decompensated heart failure. Infectious hospitalizations were defined as any hospitalization for a documented fever, bacteremia, viremia, fungemia, and pneumonia. Pathologist confirmed records were reviewed for the presence of International Society for Heart and Lung Transplant (ISHLT) defined rejection [21] (please see Table 3).

Primary endpoints were ACM, ISHLT defined rejection of 2R and greater, and hospitalizations for infectious or cardiac etiologies. Secondary endpoints were time to initial hospitalization and hospitalizations for any cause and average serum levels of tacrolimus at 6 months after transplant.

### 2.2. Data Analysis

Baseline characteristics between depressed and nondepressed patients were compared using the *χ* ^2^ test for categorical variables. Overall survival was assessed using the Kaplan-Meier method. Time to first hospitalization was assessed utilizing a modified Kaplan-Meir method. The curves were compared using a log-rank test.

Data were analyzed using SAS statistical software version 9.1 (SAS Institute Inc.). All statistical tests were 2-sided, and a probability value of less than 0.05 was used to define statistical significance.

## 3. Results

In the study period, a total of 102 patients underwent OHT. 26 patients (26%) were diagnosed with depression (80% pre-OHT and 20% post-OHT). Of the patients with depression 92% were on an antidepressant medication (79% selective serotonin reuptake inhibitor, SSRI; 13% Other antidepressants; 8% serotonin norepinephrine reuptake inhibitor, SNRI) and the remaining 8% received psychotherapy weekly. Baseline characteristics were similar (Table 1). The transplant group was predominately Caucasian (81% versus 75%, *P* = 0.53) and male (65% versus 76%, *P* = 0.23). Mean age was similar between the patients with depression versus nondepressed patients (57.6 versus 56.9, *P* = 0.79). The etiology of cardiomyopathy and LVAD use prior to OHT was similar between the two patient groups (46% versus 45% *P* = 0.92) prior to OHT.

## 4. Survival

**Figure 1:**
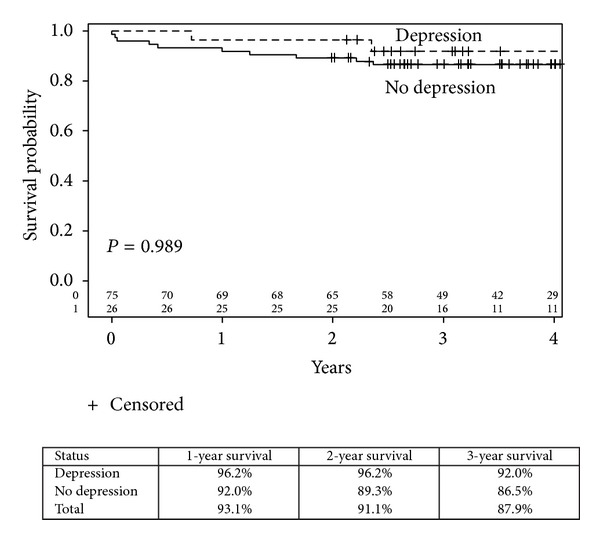
Kaplan-Meier overall survival curves by depression status (log-rank *P* value =0.989).

There was no overall statistical difference in survival over a 5-year period (Figure 1) between the two groups. There is a slight trend favoring the depressed cohort.

## 5. Rejection

As indicated in Table 2, rejection episodes and severity of rejection (ISHLT grade 2R and greater) were not statistically different between depressed and nondepressed patients. Serum tacrolimus levels were not statistically different 6 months after OHT.

## 6. Hospitalizations

**Figure 2:**
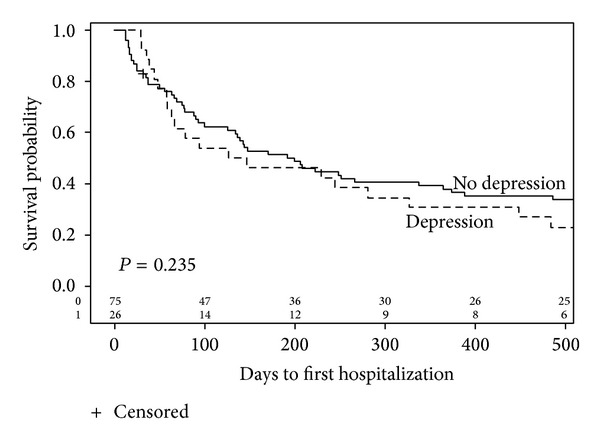
Kaplan-Meier curves for time-to-first hospitalization by depression status (log-rank *P* value =0.235).

**Figure 3:**
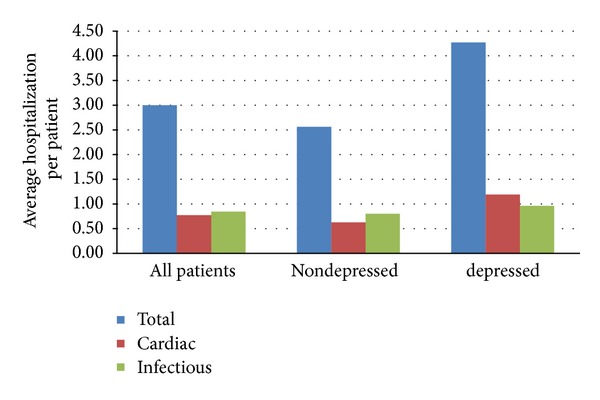
Post-heart-transplant hospitalizations. Hospitalization frequency between depressed and nondepressed patients. Depressed patients on average were hospitalized more frequently, *P* = 0.05. No statistical difference for infectious (*P* = 0.58) and cardiac hospitalizations (*P* = 0.11).

Time to first hospitalization is illustrated in Figure 2. Median time to first hospitalization was identical in both groups at 88 days. All-cause hospitalizations were higher in the depressed group versus the nondepressed group (4.3 versus 2.6 hospitalizations per patient, *P* = 0.05). There was no statistical significance between the two groups for cardiac (1.19 versus 0.63, *P* = 0.11) or infectious (0.8 versus 0.96, *P* = 0.58) hospitalizations (Figure 3).

## 7. Discussion

In our study of 102 consecutive OHT patients we found that the outcomes between patients with and without clinical depression did not differ in survival, cardiac or infectious hospitalizations, and organ rejection. Further the serum levels of immunosuppressant were similar between the groups during the follow-up period. The prevalence of depression in this cohort was 26%, with 92% of these patients receiving antidepressant medications. 80% of the depressed patients were diagnosed before OHT and the remaining 20% were diagnosed within 1 year after OHT.

Multiple studies have demonstrated an association between increased morbidity and mortality in depressed patients with concomitant cardiovascular disease compared to nondepressed patients [22–25]. Zipfel et al. in a study evaluating the effects of depression in the post-heart-transplant patient demonstrated that patients with high depression scores and an underlying etiology of ischemic cardiomyopathy were at increased risk of mortality after transplant [26]. Bush et al. underscore this concept but highlight that the presence of minimal symptoms of depression increases the risk of mortality [27]. Havik et al. demonstrated similar findings, with depression being an independent predictor of mortality in post-heart-transplant patients [28]. Studies have shown that depressed patients are more likely not to comply with prescribed medications, which would be devastating in a heart transplant patient [29–32]. Dew et al. identified specific risk factors that would predict poor post-heart-transplant outcomes in the depressed patient cohort, and they include poor social support, the use of avoidance coping strategies for health problems, and low self-esteem [33]. Dobbels et al. stress the importance of pretransplant screening, particularly focusing on self-reported nonadherence, lower social support, and education levels [34] to lower the risk of complications and organ rejection after OHT. Owen et al. in study assessing posttransplant complications followed 108 transplant recipients for approximately 3 years and identified previous suicide attempts, nonadherence to medical recommendations, and medications, alcohol, and substance abuse as risk factors for decreased survival. They further identified previous suicide attempt as an independent risk factor for posttransplant infections and speculated that early intervention may promote improved outcomes in this patient population [35].

Our results differ from the previous OHT studies in that our study population was initiated on antidepressant medications or was undergoing psychotherapy treatment either before transplant or within one year after transplant. Rogal et al. in a recent study looking at the impact of antidepressant use among patients identified with depression before liver transplant found similar findings that there was no difference in survival and organ rejection when compared to nondepressed patients [36]. Depression is one of the most common and debilitating illnesses that frequently goes undiagnosed and when diagnosed is frequently undertreated [37, 38]. Early identification and initiation of treatment of depression have been shown to improve outcomes as shown in a study by Berkman et al., where patients after myocardial infarction (MI) were initiated on treatment for depression. This study demonstrated a reduction in depressive symptoms and no difference in survival after MI between depressed and nondepressed patients [38, 39].

The ISHLT in the most recent cardiac transplant guidelines recommends routine screening for depressive symptoms [40]. Heart transplant patients are at increased risk of developing major depressive disorders because of the high incidence of depression after OHT, and regular screening and early intervention should be initiated in this patient population [41]. The Patient Health Questionnaire 9 (PHQ-9) is a self-administered diagnostic tool for common mental disorders. The PHQ-9 has been validated as a diagnostic test that can be administered in an office setting and has the advantages of objectively monitoring depressive severity and response to therapeutic management [42–44]. The PHQ-9 can be administered to patients over the phone and has been shown in a study to have similar sensitivity in identifying depression when compared to in-person screening [45].

Organ transplantation involves the allocations of scarce resources. In a large cost analysis study DiGiorgi et al. estimate the cost of initial hospitalization for OHT to be $124,830 and $6,356 for readmissions [46]. Shireman et al. estimated annual cost of graft maintenance to be $58,093 [47]. Studies have shown that untreated depression is associated with increased healthcare utilization [48–50]. Hence, based on our findings early identification and treatment of depression in OHT patients provide comparable outcomes to OHT recipients without depression and possibly less resource utilization. Heart transplant and posttransplantation care has evolved over the past half century; Bruschi et al. in a study highlight a 25-year experience of the complexities of the management of posttransplant care and how careful patient selection, clinical follow-up, and multidisciplinary teamwork involving heart failure experts and other allied health professionals led to excellent long term results [51].

The retrospective nature of our study and the sample size may limit the generalizability of these findings. However, our OHT patients obtain all of their follow-up care at a single institution, and we feel that all outcomes are adequately captured from our chart review. All patients underwent clinical evaluation by the cardiac behavioral medicine service, and depressed patients were further evaluated by the inpatient psychiatric liaison service. The diagnosis of depression was based on documentation by the treating heart failure physician after clinical evaluation by cardiac behavioral medicine and psychiatric consultation. Since the diagnosis of depression was made by different consultant psychiatrists, the interobserver variability may be a limitation of our study; however, all these patients were evaluated by the same cardiac behavioral medicine team and hence we are confident in the diagnosis. The medications used for management of depression varied and were dependent on physician preference. Hence, there is a possibility that different medications may have varying effects in the OHT population. A prospective study using an algorithmic approach to drug selection for treating depression that do not have significant interactions with the transplant medications would overcome this limitation and is worthy of future research. The retrospective nature of this study is further limited by the possibility of the underdiagnosis of depression. It is possible that patients who developed depressive symptoms following OHT were not identified and thus have been categorized as nondepressed, further supporting a prospective study that frequently assesses depressive symptoms.

In conclusion, we have shown that early identification and treatment of depression in OHT patients are associated with outcomes similar to nondepressed patients and have the potential to result in decreased resource utilization. A larger study that looks at prospectively identifying and treating depression in OHT patients with long term follow-up for outcomes is warranted.

## Figures and Tables

**Table 1:** Baseline characteristics.

Characteristics	Depressed (*N* = 26)	Nondepressed (*N* = 75)	*P* value
Age	57.6 ± 12.3	56.8 ± 14	.79
Gender	Male 65% (17)	Male 76% (58)	.0027
	Female 35% (9)	Female 23% (17)	.0027
Race			
Caucasian	81% (21)	75% (56)	.53
African American	15% (4)	15% (11)	1.00
Hispanic	4% (1)	5% (4)	.8
Asian	0%	5% (4)	.24
Ischemic cold time	183	196	.14
LVAD	46%	45%	.92
Etiology			
ICM	38%	39%	.92
DCM	42%	37%	.65
Valv	4%	5%	.83
Myo	0%	3%	.37
Chemo	12%	11%	.88
Cong	4%	1%	.32
Fam	0%	3%	.37
TCAD	0%	1%	.60

LVAD = left ventricular assist device, ICM = ischemic cardiomyopathy, DCM = dilated cardiomyopathy, Valv = valvular cardiomyopathy, Myo = myocarditis, Cong = congenital cardiomyopathy, Fam = familial cardiomyopathy, Chemo = chemotherapy induced cardiomyopathy, and TCAD = transplant coronary artery disease.

**Table 2:** Rejections episodes by depression status, *N* (%).

Rejection	Depression	No depression	*P* value
0R	26 (100.0%)	74 (100.0%)	n/a
1R	25 (96.2%)	69 (93.2%)	1.000
2R	6 (23.1%)	22 (29.7%)	0.516
1	2 (7.7%)	18 (24.0%)	
2	2 (7.7%)	3 (4.0%)	
3	0 (0.0%)	1 (1.3%)	
4	1 (3.9%)	0 (0.0%)	
5	1 (3.9%)	0 (0.0%)	
3R	1 (3.9%)	4 (5.4%)	1.000
1	1 (3.9%)	3 (4.0%)	
2	0 (0.0%)	1 (1.3%)	
4R	0 (0.0%)	0 (0.0%)	n/a

**Table 3:** International Heart and Lung Transplant Society acute rejection criteria.

Grade	Description
0R	No evidence of acute cellular rejection
1R	Mononuclear cells infiltration without or with only one focus of myocyte damage
2R	An infiltrate plus the presence of multifocal myocyte damage
3R	An infiltrate with diffuse myocyte damage and/or associated edema, hemorrhage, or vasculitis

## Abbreviations

OHT: Orthotopic heart transplant
ACM: All-cause mortality
LVAD: Left ventricular assist device
EMR: Electronic medical record
IHLT: International Heart and Lung Transplant.
